# Ameliorating Effects of Transcutaneous Electrical Acustimulation at Neiguan (PC6) and Zusanli (ST36) Acupoints Combined with Adaptive Biofeedback Training on Functional Outlet Obstruction Constipation

**DOI:** 10.1155/2020/8798974

**Published:** 2020-09-14

**Authors:** Jie Liu, Hulin Chen, Dewei Wu, Ruiling Wei, Chaolan Lv, Juan Dong, Dandan Wu, Yue Yu

**Affiliations:** ^1^Department of Gastroenterology, Affiliated Provincial Hospital, Anhui Medical University, Hefei, Anhui 230001, China; ^2^Department of Gastroenterology, The First Affiliated Hospital of USTC, Division of Life Sciences and Medicine, University of Science and Technology of China, Hefei, Anhui 230001, China; ^3^South District of Endoscopic Center, The First Affiliated Hospital of USTC, Division of Life Sciences and Medicine, University of Science and Technology of China, Hefei, Anhui 230001, China

## Abstract

**Background:**

Stimulant laxatives are still considered the most common treatment for functional outlet obstruction constipation (FOOC). However, the effectiveness of laxatives is unsatisfactory, and the long-term use of laxatives may cause certain adverse events. With this in mind, it is, however, paramount that novel complementary treatment(s) and/or other forms of alternative medicine are adequately investigated.

**Aims:**

The study aims to explore the effects and potential mechanism(s) of transcutaneous electrical acustimulation (TEA) combined with adaptive biofeedback training (ABT) on FOOC.

**Methods:**

A total of forty-five patients with FOOC were recruited and were randomly assigned to receive either Macrogol 4000 Powder (MAC, 10 g bid) (group A, *n* = 15) only, ABT + MAC + Sham-TEA (group B, *n* = 15), or TEA + ABT + MAC (group C, *n* = 15) in a six-week study. Individual patients' constipation-symptoms (PAC-SYM) and constipation-quality of life (PAC-QOL) were both assessed and scored. Serum acetylcholine (Ach) and nitric oxide (NO) were measured from drawn blood samples while individual patients' heart rate variability (HRV) was calculated at baseline and after each corresponding therapy. Anorectal manometry and balloon expulsion test were both performed before and after treatment.

**Results:**

Firstly, participants in group C had significantly lower scores of PAC-SYM, PAC-QOL, and a decreased anal defecating pressure (ADP) as compared to participants in group B (all *p* < 0.050). These results, however, suggest the TEAs effect. Secondly, the low-frequency band (LF)/(LF + HF) ratio in groups B and C were decreased as compared to group A (*p*=0.037, *p*=0.010, respectively) regarding HRV. On the other hand, the high-frequency band (HF)/(LF + HF) ratio in groups B and C showed an opposite outcome. Finally, the serum Ach in groups B and C was significantly higher as compared to group A (*p*=0.023, *p*=0.012, respectively). Of significant importance, the serum NO in groups B and C were notably low as compared to group A (*p*=0.001, *p* < 0.001, respectively).

**Conclusions:**

TEA, combined with ABT, effectively improves constipation symptoms as well as QOL in FOOC patients. It is, however, achieved by decreasing ADP, which mechanisms are mediated via the autonomic and enteric mechanisms.

## 1. Introduction

Functional outlet obstruction constipation (FOOC) is painful defecation caused by pelvic floor muscles (internal anal sphincter or the striated muscles) not relaxing or paradoxical contraction of the external anal sphincter and the puborectalis muscles [1–3]. It is estimated that more than 17% of the global population will suffer from chronic constipation, with up to 50% of patients referred to tertiary centers for the management of chronic constipation being diagnosed with outlet obstruction constipation OOC [4, 5]. Findings by Martelli et al. [6] have shown that central and peripheral neurological disorders are responsible for FOOC, which leads to dysfunctional defecation. Currently, stimulant laxatives are the most common treatment for FOOC. However, laxatives are not able to alleviate all the symptoms and long-term use of laxatives may have adverse effects [7]. Therefore, the development of novel complementary treatment(s) and other forms of alternative medicine is vital.

Adaptive biofeedback training (ABT), an improvement of traditional biofeedback, enables patients to adapt and adjust gradually with regard to anorectal parameters using anorectal manometry [8]. ABT helps in providing visual and auditory feedback using pressure-sensor technology. The ABT aid enables patients to adequately coordinate the muscular functions of the pelvic floor. The aid practically teaches the patient to defecate by relaxing the pelvic floor muscles while simultaneously applying adequate propulsive force toward the rectum and anal canal. However, the effectiveness of ABT in the treatment of FOOC is limited, and a part of patients can not obtain symptoms improvement [9]. Therefore, novel complementary treatment(s) and/or other forms of alternative medicine are adequately investigated.

Acupuncture, an integral part of traditional Chinese medicine, has been used to treat functional gastrointestinal diseases (FGID) in China for decades. Acupuncture points (acupoints), such as Neiguan (PC6) and Zusanli (ST36), are commonly used to treat FGIDs, including functional constipation [10, 11]. Studies on PC6 and ST36 acupoints have reported successful alleviation of abdominal symptoms, especially in improving constipation symptoms [12–14]. Transcutaneous electrical acustimulation (TEA), a noninvasive and needleless method (technology), is widely accepted by patients [15]. Preliminary studies of TEA using ST36 and PC6 acupoints have indicated successful alleviation of constipation symptoms and an improved intestinal symptom, particularly in slow-transit constipation [11]. In addition, TEA is reported to improve dyspeptic symptoms by enhancing vagus nerve activity while inhibiting the activities of the sympathetic nerve [16]. Unfortunately, there are no reports on its mechanism(s) and potential effects on FOOC.

Although the use of TEA or ABT alone has been reported to improve constipation symptoms [10, 17], it is not known whether a combination of both would have a synergistic effect for the treatment of FOOC. Therefore, we designed this study to (i) explore the possible synergistic effect(s) of combining TEA and ABT, (ii) determine its anorectal motility in FOOC, and (iii) determine the associated potential autonomic mechanisms in FOOC patients.

## 2. Methods

### 2.1. Patients

Patients (outpatients and inpatients) who met the Rome IV diagnostic criteria [18] for FOOC were diagnosed and recruited into the study at the Department of Gastroenterology, Anhui Provincial Hospital from November 2016 to January 2020. The following inclusion criteria were used: (i) patients with chronic symptoms of strain, incomplete evacuation, hard stools, anorectal obstruction, manual maneuvers, and weak defecation movements <3 times per week, (ii) paradoxical or nonrelaxing puborectalis muscle observed during at least two individual studies such as physical exam, anorectal manometry, defecography, and balloon expulsion test (BET), and (iii) signs of abnormal anal defecating pressure [19, 20]. The exclusion criteria included age >70 years, pregnant and lactating patients, severe heart and lung diseases, diabetes, nephropathy, and other chronic diseases associated with gastrointestinal disorders such as inflammatory bowel disease, ulcers, cancer, and esophageal varices. Patients were also excluded if they had slow-transit constipation diagnosed using colonic transit radiopaque markers.

The protocol used in the study was approved by the Ethics Committee of Anhui Provincial Hospital (Registration No: 2016 L36). And this study protocol was registered on the Chinese Clinical Trial Registry (No. ChiCTR-2000037449). Written informed consent was obtained from all participants before their inclusion in the study.

### 2.2. Selection of Acupoints

Traditional Chinese medicine (TCM) history indicates that ST36 was used to invigorate functions of the stomach and intestine, including functional constipation [10, 11] while PC6 was used to nourish the heart, thereby restoring a balance in the mind. In addition, PC6 was used to restore liver function for patients with obvious depression, anxiety, or insomnia symptoms [14]. A combination of ST36 and PC6 helps in restoring the normal “qi” flow inside the stomach and spleen meridian. Studies on PC6 or ST36 acupoints have reported successful alleviation of abdominal symptoms, including improvement of constipation symptoms [12–14]. These therapeutic principles enhanced our decision to select ST36 and PC6 acupoints for use in this study. Results obtained from this study will add further credence to the use of these acupoints.

### 2.3. Experimental Protocol

This study is a prospective trial with a randomized control and single-blind design. Eligible patients were randomly divided into three groups (A, B, and C) based on random numbers. Patients in group A (*n* = 15) were treated with Macrogol 4000 Powder (MAC, 10 g) twice a day. Group B patients (*n* = 15) were treated with ABT (twice a day) + MAC 10 g (twice a day) + Sham-TEA (twice a day). On the other hand, group C patients (*n* = 15) received TEA for 30 minutes (twice a day) + ABT (twice a day) + MAC 10 g (twice a day). All the patients were treated for six weeks. No statistical differences were observed among the three groups in terms of age, gender, BMI, and the duration of constipation (Table 1).

Patients were enrolled for ABT practice at the Anhui Provincial Hospital Gastroenterology Department using the Med Kinetic (Ningbo Maida Medical Device Inc. Ningbo, China) software. Patients were also instructed to practice squeezing and relaxing maneuvers for 30 minutes < 3 times a week. The patients were encouraged to practice the maneuvers twice a day when they were at home.

A watch-size stimulator (Med Kinetic, Ningbo, China), which can be attached to the arm and leg using an electrode piece, was used for TEA at Zusanli (ST36) and Neiguan (PC6) acupoints. The watch-size stimulator was maintained at 25 Hz (5 mA), for 30 minutes twice a day, with the stimulation parameters being programmed using a specific computer. The same parameters were used for Sham-TEA which was performed at a nonchannel and noncollateral nonacupoints 2 cm away from ST36 and PC6 acupoints [21].

### 2.4. Measurements

#### 2.4.1. Adaptive Biofeedback Training

Adaptive biofeedback training (ABT) was done using an anorectal manometry system with an 8-channel catheter and a balloon attached to the tip. The operation was conducted by a therapist who had specialized in the field. The therapist first provided individualized education to each patient about the structure of the anus and rectum, defecation mechanism(s), gastrocolic reflex, constipation, and the concept of ABT. Secondly, a catheter was inserted into the anal canal of the patients, with each patient able to view the pressure pattern recordings on a computer. Patients were then instructed to observe the pressure changes, particularly the anal sphincter response during squeezing and straining. They were also taught how to relax their anal sphincter while simultaneously observing the pressure changes. Finally, patients began biofeedback training using the software by Med Kinetic (Ningbo Maida Medical Device Inc. Ningbo, China). The patients watched and listened to instructions from the computer on how to coordinate the movement of anorectal and abdominal muscles [22].

#### 2.4.2. Balloon Expulsion Test (BET)

A 3.5 cm latex balloon with 50 mL of warm water was placed in the rectum using a tube with a diameter of 3 mm attached to a balloon. After activating a stopwatch, the therapist left the operation room to provide privacy for the patient during the balloon expulsion test. The patient was then instructed to excrete the balloon into a toilet and stop the timing. All attempts were recorded with expulsion failure being defined as when patients were unable to expel the intrarectal balloon in 1 minute [23].

#### 2.4.3. High Anorectal Resolution Manometry (HARM)

All participants received a HARM test (Med Kinetic, Ningbo, China) at the beginning and the end of each therapy session. Sodium phosphate solutions were prepared for bowel evaluations before the anorectal manometric evaluation was done. Anal sphincter pressure was measured using a water-perfused anorectal manometric catheter with eight pressure sensors at 1 cm interval. The device adopts proprietary pressure transduction technology allowing each pressure-sensor element to detect pressure over a length of 2.5 mm in each of the 12 radially dispersed sectors. An anorectal catheter was inserted in each patient's rectum and the patient was instructed to perform a simulated defecation process for 20 seconds. The anal defecating pressure (ADP) for each patient was recorded using the ManoView software [23].

#### 2.4.4. Questionnaire

Two self-report questionnaires, Patients Assessment of Constipation-Symptoms (PAC-SYM) [24] and the Patients Assessment of Constipation-Quality of Life (PAC-QOL) [25], were used to assess the severity of reported chronic constipation symptoms and the quality of life of patients. PAC-SYM is a 12-item instrument developed for analyzing the severity of chronic constipation symptoms. The questionnaire is split into three dimensions; abdominal, rectal, and stool. Various items are scored on a 5-point Likert scale ranging from 0 (absence of symptoms) to 4 (very severe). The higher the total score, the more severe the patient constipation symptoms. The scale was further used to evaluate the response of each patient to the administered chronic constipation treatment options. On the other hand, PAC-QOL is a 28-item instrument developed to assess the influence of constipation on a patient's daily quality of life (QOL) using four dimensions (physical discomfort, anxiety, psychological discomfort level, and satisfaction). The various items of PAC-QOL are scored on a 5-point Likert response scale, ranging from 0 (Not at all) to 4 (Extremely). The total score is positively correlated to the QOL of the patients, where a high total score indicates a more serious impact of constipation on the patient's QOL.

#### 2.4.5. Assessment of Autonomic Functions

The autonomic nervous functions of participants were evaluated using spectral analysis of heart rate variability (HRV). HRV analysis software (Cardiotrak Holter system version: 1.2.0.0, Hangzhou Baihui Electrocardiograms, China) was used to process each patient's HRV data, while an electrocardiogram (ECG) recording (ct-082, Hangzhou Baihui Electrocardiograms, China) was used to provide HRV signals. Power spectral analysis was then conducted, followed by calculation of the power in each frequency subband [26]. The power in the low-frequency band (0.04–0.15 Hz, LF) mainly represents sympathetic activity while the power in the high-frequency band (0.15–0.50 Hz, HF) represents parasympathetic or vagal activities. In addition, the power obtained using the LF/(HF + LF) ratio represents sympathetic activity, while that of the HF/(HF + LF) ratio represents parasympathetic activity.

#### 2.4.6. Ach and NO Assays

Blood samples were collected before and after treatment in a fasting state and placed in chilled EDTA and aprotinin tubes. The samples were then centrifuged at 4200 g and 4°C for 10 min and stored at 4°C until extraction was done. Serum Ach contents and NO levels were determined using corresponding commercial ELISA kits (Jiancheng Institute of Biology and Technology, Nanjing, China; NO product batch number: 20170113; Ach products batch number: 20170210).

#### 2.4.7. Statistical Analysis

SPSS16.0 statistical software was used for data analysis. Continuous variables were presented as mean ± standard deviation, while the Chi-squared test was used for comparison of discontinuous parameters (baseline data and BET). Multiple parametric groups were compared using a 1-way ANOVA test or nonparametric test, while pre- and posttreatment data were analyzed using a paired *t*-test. Statistical significance was determined at a *p* value of 0.05 or less.

## 3. Results

**Figure 1:**
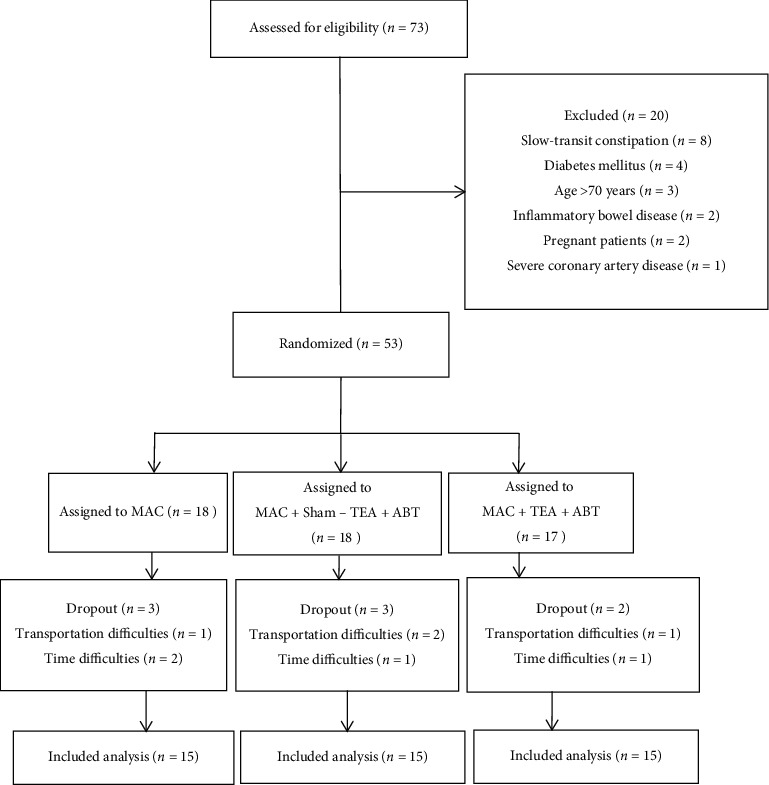
Flow chart of study participation.

A total of 73 patients were eligible for the study. Due to various reasons, 20 patients were excluded from the study. The remaining 53 patients were randomly assigned to three groups based on the random numbers which were generated by the central randomization system. 45 patients completed the study while eight patients (three from group A and B, respectively; two from group C) dropped out from the study (dropout rate of group A and B: 16.67%; dropout rate of group C: 11.76%) due to time and transportation difficulties. The consort flowchart is shown in Figure 1.

### 3.1. The Effects of Treatments on Constipation Symptoms

**Figure 2:**
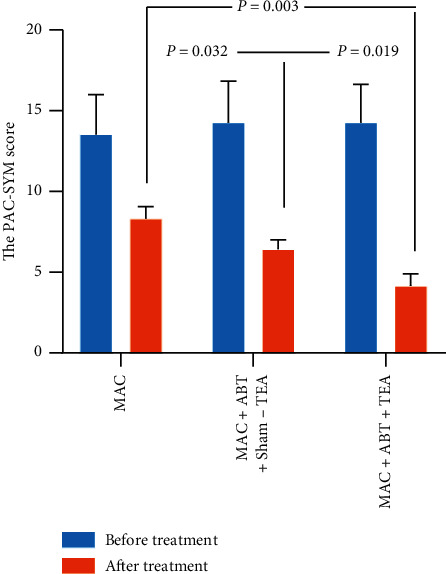
The comparison of the PAC-SYM score pre- and posttreatment among groups. The symptom score of three groups decreased after the corresponding treatment (^*∗*^*p* < 0.050). There was a significant difference between ABT + MAC + Sham-TEA and ABT + MAC + TEA treatment (*p* < 0.050).

According to results, the combination therapy of TEA and ABT is more effective in improving symptoms of FOOC when compared with ABT treatment alone. PAC-SYM score for all groups significantly declined after each of the three treatments (*p* < 0.050) with the treatment effect in group C being the most potent (Figure 2). Group C had a lower PAC-SYM score when compared to group B (4.16 ± 0.73 vs. 6.43 ± 0.58, *p*=0.019), suggesting a net TEA effect. Group C also had a lower PAC-SYM score than group A (4.16 ± 0.73 vs. 8.33 ± 0.72, *p*=0.003), suggesting a combined effect of TEA and DBT. There was a significant difference between group A and group B in the symptom score after treatment (8.33 ± 0.72 vs. 6.43 ± 0.58, *p*=0.032), suggesting a significant therapeutic effect of sham-TEA combined with DBT treatment.

### 3.2. Effects of Treatments on Constipation-Quality of Life

**Figure 3:**
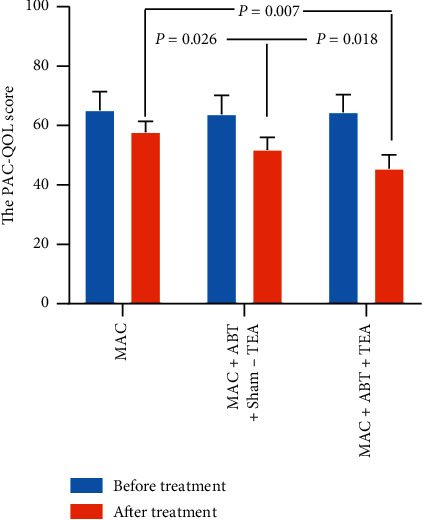
The comparison of the PAC-QOL score pre- and posttreatment among groups. The quality of life score of three groups decreased after treatment (^*∗*^*p* < 0.050). There was a significant difference between ABT + MAC + Sham-TEA and ABT + MAC + TEA treatment (*p* < 0.050).

Results indicate that TEA combined with ABT is more effective than ABT alone in improving the quality of life of FOOC patients. The PAC-QOL score in all groups was significantly decreased after each of the three treatments (*p* < 0.050) with group C treatment effect being the most potent (Figure 3). This is because the PAC-QOL score of group C was lower than that of group B (45.57 ± 4.53 vs. 51.86 ± 4.21, *p*=0.018) suggesting a net TEA effect, and also lower than that of group A (45.57 ± 4.53 vs. 57.86 ± 3.57, *p*=0.007), suggesting a combined TEA and ABT effect. A significant difference was also noted in the quality of life score when the treatment effects between group B and group A were compared (51.86 ± 4.21 vs. 57.86 ± 3.57, *p*=0.026), suggesting a significant therapeutic effect after treatment with sham-TEA combined with ABT.

### 3.3. Effects of Treatments on ADP

**Figure 4:**
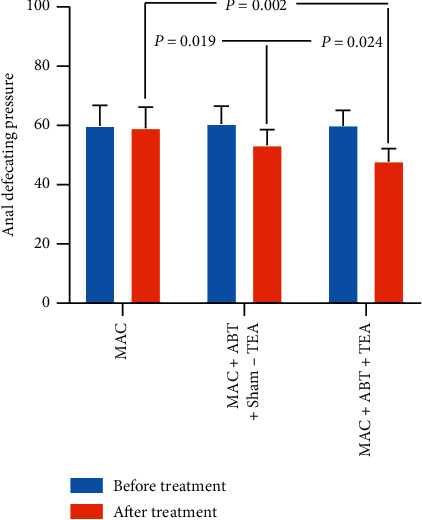
The ADP of groups B and C significantly decreased before and after the treatment (^*∗*^*p* < 0.050). There was a significant difference between MAC + ABT + TEA and MAC treatment (*p* < 0.050). There was also a significant difference between sham-TEA and TEA treatment (*p* < 0.050), and TEA group treatment decreased ADP lower.

Results indicated that the combination of TEA and ABT and ABT alone had a significant decrease in ADP. There was no change of the ADP in group A after MAC treatment (*p*=1.0), while a significant decrease was noted in group B (*p* < 0.050) and group C (*p* < 0.010) (Figure 4). In addition, the ADP in group C was significantly lower than that in group B (47.89 ± 4.26 vs. 53.27 ± 5.23, *p*=0.024) and group A (47.89 ± 4.26 vs. 59.14 ± 7.06, *p*=0.002). There was a significant difference in ADP after treatment when group A was compared with group B (59.14 ± 7.06 vs. 53.27 ± 5.23, *p*=0.019). The results suggest that both TEA and ABT lead to ADP decline with a combination of both being the most potent.

### 3.4. Effects of Treatments on BET

A combination of TEA and ABT significantly promoted balloon expulsion in the patients. Ten patients in group A, nine in group B, and ten in group C showed abnormal BETs before treatment. After treatment, nine patients in group A, four in group B, and three in group C showed abnormal BETs. There was no change in BET for patients in group A (*p*=0.705) and group B (*p*=0.065) after MAC and MAC + ABT + sham-TEA treatments, respectively. However, there was a significant decrease in group C (*p*=0.010) with results suggesting that TEA combined with ABT helped the patients' balloon expulsion.

### 3.5. Mechanisms Involving Autonomic Functions

**Figure 5:**
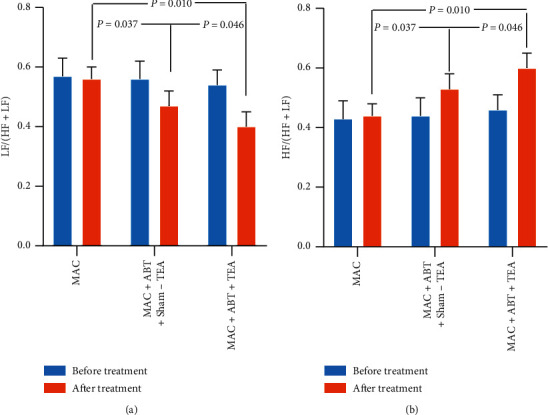
(a). The ratio of LF/(LF + HF) of groups B and C significantly decreased before and after treatment (^*∗*^*p* < 0.050). After treatment, there was a significant difference between ABT + MAC + Sham-TEA and ABT + MAC + TEA treatment (*p* < 0.050). (b). The ratio of HF/(LF + HF) of groups B and C significantly increased before and after treatment (^*∗*^*p* < 0.050). After treatment, there was a significant difference between ABT + MAC + Sham-TEA and ABT + MAC + TEA treatment (*p* < 0.050).

Spectral analysis of HRV indicated that both TEA and ABT significantly enhanced vagal activity while inhibiting sympathetic activity at the same time. MAC treatment did not alter LF/(LF + HF) or HF/(LF + HF) (*p*=0.403) (Figures 5(a) and 5(b)). The ratio of LF/(LF + HF) in group A was significantly higher than that in group B (0.47 ± 0.05 vs. 0.56 ± 0.04, *p*=0.037) and group C (0.40 ± 0.05 vs. 0.56 ± 0.04, *p*=0.010). Conversely, the ratio of HF/(LF + HF) in group A was significantly lower than that in group B (0.53 ± 0.05 vs. 0.44 ± 0.04, *p*=0.037) and group C (0.60 ± 0.05 vs. 0.44 ± 0.04, *p*=0.010).

### 3.6. Mechanisms Involving Enteric Neurotransmitters

**Figure 6:**
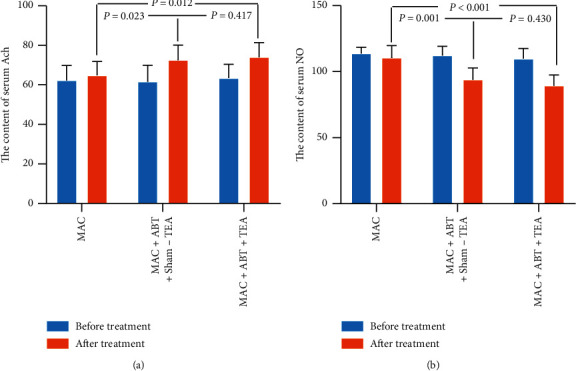
(a). The content of serum Ach of groups B and C significantly increased before and after treatment (^*∗*^*p* < 0.050). After treatment, there was a significant difference between MAC + ABT + TEA and MAC treatment (*p* < 0.050), and there was also a significant difference between MAC + ABT + sham-TEA and MAC treatment (*p* < 0.050). (b). The content of serum NO of groups B and C significantly decreased before and after treatment (^*∗*^*p* < 0.050). After treatment, there was a significant difference between MAC + ABT + TEA and MAC treatment (*p* < 0.050), and there was also a significant difference between MAC + ABT + sham-TEA and MAC treatment (*p* < 0.050).

Results indicate that both TEA and ABT enhance the release of Ach. The level of serum Ach was significantly increased after the treatment in group B (61.59 ± 8.32 vs. 72.58 ± 7.56, *p* < 0.001) and group C (63.47 ± 6.98 vs. 74.04 ± 7.31, *p* < 0.001) (Figure 6(a)). There was no change in serum Ach levels after MAC treatment in group A (62.32 ± 7.46 vs. 64.78 ± 6.98, *p*=0.174). After six weeks of treatment, the level of serum Ach in group A was significantly lower than that of group C (64.78 ± 6.98 vs. 74.04 ± 7.31, *p*=0.012) and group B (64.78 ± 6.98 vs. 72.58 ± 7.56, *p*=0.023). There was no statistical difference between group B and group C serum Ach levels (72.58 ± 7.56 vs. 74.04 ± 7.31, *p*=0.417).

According to obtained results, TEA and ABT significantly inhibited the release of NO. Serum NO level was significantly reduced after treatment in group B (112.29 ± 6.89 vs. 93.95 ± 8.83, *p* < 0.001) and group C (109.78 ± 7.75 vs. 89.45 ± 7.96, *p* < 0.001) (Figure 6(b)). On the other hand, there was no change in the level of serum NO in group A after MAC treatment (113.86 ± 4.58 vs. 110.49 ± 9.14, *p*=0.098). After six weeks of therapy, the level of NO in group C and group B was significantly lower than that in group A (89.45 ± 7.96 vs. 110.49 ± 9.14, *p* < 0.001; 93.95 ± 8.83 vs. 110.49 ± 9.14, *p*=0.001). There was no statistical difference between group B and group C NO levels (93.95 ± 8.83 vs. 89.45 ± 7.96, *p*=0.430).

## 4. Discussion

In the current study, after six weeks of TEA combined with ABT treatment, the PAC-SYM scores and the PAC-QOL scores were significantly lower than that of MAC alone and MAC + ABT + sham-TEA treatment. Meanwhile, ADP was also significantly higher than that of MAC alone and MAC + ABT + sham-TEA. FOOC is characterized by the lack of coordination between the abdominal and pelvic floor muscles during defecation [27]. Statistics obtained from recent research [28] indicate that the prevalence value of functional constipation (FC) is 7.8% with regard to the Rome IV diagnostic criteria. Lee et al. [4], and Choung et al. [5] reported that over 50% of patients diagnosed with chronic constipation have associated outlet obstruction. Administration of laxatives to patients does not alleviate several symptoms, thereby posing severe health challenges to patients and a tremendous burden on the society at large [29]. Dyssynergic defecation, enteric nervous dysfunctions, and autonomic dysfunctions play major roles in FOOC. Therefore, improving autonomic nervous activities and coordinating pelvic floor muscles are considered adequate complementary treatment methods [16].

A previous study has shown that ABT is straightforward and more effective when compared to the traditional way [8]. In this study, adaptive biofeedback training (ABT) using pressure recordings was used to recover the regular defecation pattern. While defecating, patients were instructed to practice relaxing their anal sphincter while coordinating pelvic floor muscles to help apply adequate propulsive abdominal force toward the rectum and anal canal [30, 31]. Biofeedback training significantly improves constipation symptoms such as defecation difficulties, hard stools, and straining [17]. What is more, Xu et al. [32] reported significant improvement in symptoms when ABF was compared to traditional biofeedback training. Nonetheless, recent studies indicated that a combination of biofeedback training and correct diet can be used as complementary medicine for patients with obstructed defecation, thereby improving clinical symptoms of constipation [20, 33].

TEA is a widely accepted treatment method since it can be self-administrated by patients. It also has the advantage of being a noninvasive needleless method without obvious side effects [20]. Our research shows that PAC-SYM and PAC-QOL scores were significantly lower after six weeks of TEA combined with ABT treatment than for MAC alone and MAC + ABT + sham-TEA treatments. This is consistent with reports from previous studies of PC6 and ST36 acupoints which have shown TEA to be effective in ameliorating constipation symptoms, improving anxiety and depressive state in patients, while accelerating colonic transit in slow-transit constipation [11]. A meta-analysis study reported that electrical acustimulation improves spontaneous bowel movements, thereby reducing FC symptoms [34]. In addition, Zhu et al. [35] reported that electrical acustimulation in patients with FC was effective in increasing gastrointestinal transit and improving defecation frequency. Most importantly, this study emphasized that transcutaneous electrical acustimulation has a better curative effect and synergistic effect.

Gastrointestinal physiological functions play an essential role in FOOC. However, its role mainly depends on the regulative effects of CNS, ANS, and ENS. In this study, the autonomic cardiac function was evaluated as a surrogate for autonomic nerve function using the spectral HRV analysis. The method has previously been used to show that a sympathetic or parasympathetic postprandial ratio increases with a decrease in vagal activity [36]. Changes in cardiac autonomic functions induced by meals suggest that it can also represent gastrointestinal autonomic function [37]. Interestingly, Liu et al. reported that an increase in vagal activity is associated with constipation [16]. In addition, a study on TEA showed that ST36 acupoints could affect the balance between sympathetic and parasympathetic nerves, thereby significantly decreasing sympathetic activities while simultaneously enhancing vagal tone [10]. Results from this study indicate similar outcomes where a combination of TEA and ABT increased vagal or parasympathetic activity while simultaneously suppressing sympathetic activity. This might have led to well-coordinated pelvic floor muscles.

Ach plays a vital role in peristalsis and accelerating gastrointestinal smooth muscle contraction. The level of Ach is also associated with vagus nerve activity. In this study, the level of Ach in patients who received TEA combined with ABT treatment was significantly higher than that in the other two groups, suggesting that increased Ach might be associated with promoting intestinal peristalsis and promoting the expulsion of feces. Our findings are consistent with Huang et al. [38] who reported that sacral nerve stimulation (SNS) could improve constipation symptoms through enhancing distal colon motility and stimulating the release of Ach in blood. SNS also significantly enhances vagal activities and inhibits sympathetic tones. On the other hand, NO is an inhibitory neurotransmitter in the enteric nervous system associated with gastrointestinal smooth muscle relaxation. Liang et al. [39] reported that electric acupuncture was an effective therapy for model mice with constipation through downregulating the release of NO. Results from this study indicate that the level of NO in patients treated with TEA + ABT was significantly lower than those in the MAC alone and the MAC + ABT + sham-TEA group. On the other hand, there were no significant changes between the MAC alone and MAC + ABT + sham-TEA group. The results suggest that NO may be associated with enhanced intestinal motility for promoting excretion of intestinal contents. The authenticity of the enteric mechanism was enhanced by the results of the balloon expulsion test, which indicated that TEA combined with ABT promoted patients' balloon expulsion.

Results from this study show that a combination of TEA and ABT significantly decreases ADP, promotes balloon expulsion in the patients, and improves clinical manifestations of FOOC. In addition, the combination also significantly increases vagal activity while simultaneously inhibiting sympathetic activity. With regard to enteric neurotransmitters, the combination of TEA and ABT significantly enhances the release of Ach while suppressing NO. Although ABT and MAC also improved FOOC, the combined treatment with TEA was the most potent in almost every aspect of the parameters studied. This is consistent with the popular opinion that constipation improvement is attributed to a decrease in ADP, mediated via autonomic and enteric mechanisms.

## 5. Limitations

This study is limited by the fact that it is a single-blind research because it is difficult to conduct double-blinding in acupuncture studies. The TEA group was judged according to the position of stimulating acupoints; thus, the cofounding factor of the physician's judgment cannot be ruled out, thereby affecting the subjectivity of this study. In addition, the study was done at one clinical center using a relatively small sample. Therefore, the results of the study may not reflect the same effect of TEA treatments on all FOOC patients. In addition, patients who dropped out were not included in the analysis of results, which may affect the credence of the results. Further research using large-scale, multicenter, double-blind, randomized placebo-controlled trials should be done to better determine the response of patients with FOOC after TEA treatment.

## 6. Conclusion

This study shows that TEA at Neiguan (PC6) and Zusanli (ST36) acupoints combined with ABT may effectively alleviate symptoms of FOOC in patients by decreasing ADP. Although ABT and MAC also improved FOOC, the combined treatment of TEA was most potent in almost every aspect of the parameters studied. According to our research, the ameliorating effects may be associated with autonomic and enteric mechanisms.

## Figures and Tables

**Table 1:** Baseline characteristics for the three study groups.

	Overall (*N* = 45)	Group A (*n* = 15)	Group B (*n* = 15)	Group C (*n* = 15)	F	*p*
Gender						
Male (*n*)	11	4	4	3		0.887
Female (*n*)	34	11	11	12		
Age (yeras; mean ± SE)	45.71 ± 10.64	47.20 ± 10.98	45.38 ± 12.79	43.24 ± 9.22	2.014	0.251
BMI (kg/m^2^; mean ± SE)	21.31 ± 4.03	21.53 ± 3.67	20.45 ± 3.05	21.68 ± 4.83	1.415	0.977
Duration of constipation (months; mean ± SE)	47.95 ± 7.36	43.46 ± 8.59	48.34 ± 11.39	52.48 ± 9.38	2.869	0.168

Participants in group A were treated with Macrogol 4000 Powder (MAC, 10 g, twice daily), group B with ABT (twice daily) + MAC (10 g, twice daily) + Sham-TEA (twice daily), and group C received TEA (30 min twice daily) + ABT (twice daily) + MAC (10 g, twice daily). The Chi-squared test was used for comparison of gender between the three groups and the result showed that Chi-squared = 0.241; *p*=0.887. No statistically significant difference was noted in age, gender, BMI, and the duration of constipation.

## Data Availability

All patients joined the study with written informed consent for the research use of their survey data. The data used to support the findings of this study are available from the corresponding author upon request. Data were collected by authorized researchers.
